# Supplementary material for: The effect of intrapartum prolonged oxygen exposure on fetal metabolic status: secondary analysis from a randomized controlled trial
Source: Front Endocrinol (Lausanne). 2023 Jun 27;14:1204956. doi: 10.3389/fendo.2023.1204956 (PMC10335765; doi:10.3389/fendo.2023.1204956)
Supplement: Supplementary file 3 [file DataSheet_3.zip › Result-X101SC21092977-Z01-J001-B1-42/4.MetDiffAnalysis/O.vs.A/O.vs.A_neg_zscore.pdf]

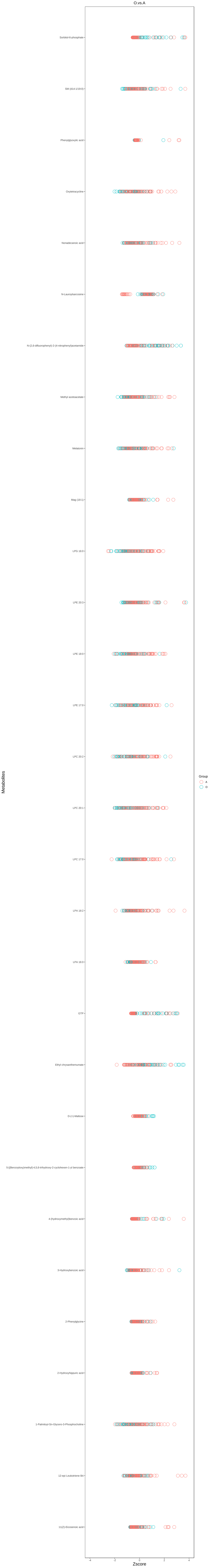
O.vs.A
Sorbitol-6-phosphate
SM (d14:1/19:0)
Phenylglyoxylic acid
Oxytetracycline
Nonadecanoic acid
N-Lauroylsarcosine
N-(2,6-difluorophenyl)-2-(4-nitrophenyl)acetamide
Methyl acetoacetate
Melatonin
Mag (18:1)
LPG 16:0
LPE 20:3
LPE 18:0
LPE 17:0
LPC 20:2
LPC 20:1
LPC 17:0
LPA 18:2
LPA 16:0
GTP
Ethyl chrysanthemumate
D-(+)-Maltose
5-[(Benzoyloxy)methyl]-4,5,6-trihydroxy-2-cyclohexen-1-yl benzoate
4-(hydroxymethyl)benzoic acid
3-Hydroxybenzoic acid
2-Phenylglycine
2-Hydroxyhippuric acid
1-Palmitoyl-Sn-Glycero-3-Phosphocholine
12-epi Leukotriene B4
11(Z)-Eicosenoic acid
Metabolites
-4
-2
0
2
4
Zscore
Group
A
O
